# Supplementary material for: Transcriptome Analysis Reveals Vimentin-Induced Disruption of Cell–Cell Associations Augments Breast Cancer Cell Migration
Source: Cells. 2022 Dec 13;11(24):4035. doi: 10.3390/cells11244035 (PMC9776984; doi:10.3390/cells11244035)
Supplement: Supplementary file 1 [file cells-11-04035-s001.zip › DEGs and enrichment analysis.zip!/DEGs and enrichment analysis/WT_VIMvsVector KEGG/ALL/WT_VIMvsVector_ALL.pathway_map.zip!/WT_VIMvsVector_ALL.pathway_map/src/hsa04914.html]

hsa04914


- hsa:107

- Up regulated genes

ENSG00000164742(1.87205666251591)

- hsa:10459

- Up regulated genes

ENSG00000116670(1.93023390989801)

- hsa:993

- Up regulated genes

ENSG00000164045(1.78085789611929)

- hsa:5347

- Up regulated genes

ENSG00000166851(2.38554234415561)

- hsa:9133

- Up regulated genes

ENSG00000157456(1.60409075038332)

- hsa:9088

- Up regulated genes

ENSG00000127564(2.77874383365084)

- hsa:890

- Up regulated genes

ENSG00000145386(1.72013468925064)

- hsa:5241

- Up regulated genes

ENSG00000082175(4.22083564138011)

Close
